# Dietary lignans, plasma enterolactone levels, and metabolic risk in men: exploring the role of the gut microbiome

**DOI:** 10.1186/s12866-022-02495-0

**Published:** 2022-03-29

**Authors:** Yanping Li, Fenglei Wang, Jun Li, Kerry L. Ivey, Jeremy E. Wilkinson, Dong D. Wang, Ruifeng Li, Gang Liu, Heather A. Eliassen, Andrew T. Chan, Clary B. Clish, Curtis Huttenhower, Frank B. Hu, Qi Sun, Eric B. Rimm

**Affiliations:** 1 Department of Nutrition, Harvard T.H. Chan School of Public Health, 665 Huntington Avenue, Boston, MA 02115 USA; 2 Microbiome and Host Health Programme, South Australian Health and Medical Research Institute, North Terrace, Adelaide, SA 5000 Australia; 3 Department of Nutrition and Dietetics, College of Nursing and Health Sciences, Flinders University, Adelaide, Australia; 4 Department of Biostatistics, Harvard T.H. Chan School of Public Health, Boston, MA USA; 5 Channing Division of Network Medicine, Department of Medicine, Brigham and Women’s Hospital, Harvard Medical School, Boston, MA USA; 6 Department of Epidemiology, Harvard T.H. Chan School of Public Health, Boston, MA USA; 7 Department of Immunology and Infectious Diseases, Harvard T.H. Chan School of Public Health, Boston, MA USA; 8 Broad Institute of Massachusetts Institute of Technology and Harvard, Cambridge, MA USA; 9 Clinical and Translational Epidemiology Unit, Massachusetts General Hospital and Harvard Medical School, Boston, MA USA

**Keywords:** Lignan, Enterolactone, Microbiome, Metabolic, Metabolites

## Abstract

**Background:**

The conversion of plant lignans to bioactive enterolignans in the gastrointestinal tract is mediated through microbial processing. The goal of this study was to examine the relationships between lignan intake, plasma enterolactone concentrations, gut microbiome composition, and metabolic risk in free-living male adults.

**Results:**

In 303 men participating in the Men’s Lifestyle Validation Study (MLVS), lignan intake was assessed using two sets of 7-day diet records, and gut microbiome was profiled through shotgun sequencing of up to 2 pairs of fecal samples (*n* = 911). A score was calculated to summarize the abundance of bacteria species that were significantly associated with plasma enterolactone levels. Of the 138 filtered species, plasma enterolactone levels were significantly associated with the relative abundances of 18 species at FDR < 0.05 level. Per SD increment of lignan intake was associated with 20.7 nM (SEM: 2.3 nM) higher enterolactone concentrations among participants with a higher species score, whereas the corresponding estimate was 4.0 nM (SEM: 1.7 nM) among participants with a lower species score (*P* for interaction < 0.001). A total of 12 plasma metabolites were also significantly associated with these enterolactone-predicting species. Of the association between lignan intake and metabolic risk, 19.8% (95%CI: 7.3%-43.6%) was explained by the species score alone, 54.5% (95%CI: 21.8%-83.7%) by both species score and enterolactone levels, and 79.8% (95%CI: 17.7%-98.6%) by further considering the 12 plasma metabolites.

**Conclusion:**

We identified multiple gut bacteria species that were enriched or depleted at higher plasma levels of enterolactone in men. These species jointly modified the associations of lignan intake with plasma enterolactone levels and explained the majority of association between lignan intake and metabolic risk along with enterolactone levels and certain plasma metabolites.

**Supplementary Information:**

The online version contains supplementary material available at 10.1186/s12866-022-02495-0.

## Background

Lignans are a class of secondary plant metabolites that are part of the large polyphenol family [1]. Lignans are particularly rich in flax and sesame seeds, although whole grains, other seeds, legumes, coffee, and wine, account for the primary lignan intake in the general population [2]. Both animal [3, 4] and human experiments [5–7] have demonstrated potentially beneficial effects of lignan intake on human health, yet the biological mechanisms are not fully understood. It is well elucidated that plant lignans are converted by gut microbiota to produce more bioactive enterolignans [8–11], which are readily absorbed into the circulation and exert subsequent health effects. In this process, the biosynthetic capacity of the gut microbiota to metabolize plant lignans[12, 13] includes *O*-deglycosylation of plant lignans to the aglycone form of lignans and the subsequent *O*-demythylation, dehydroxylation, and dehydrogenation of aglycones to produce enterolactone and/or enterodiol [14]. Several gut microbiota species involved in lignan metabolism, including *Ruminococcus bromii*, *Bacteroides ovatus*, and *Eggerthella lenta*, were identified through in vitro experiments [15–20].

Early culture-based studies, although critical to establish bacteria-mediated capacity, may have not considered all microbes that contribute to the production of enterolignans due to the known difficulties in culturing many of the microbes comprising the human gastrointestinal microbiome. The use of metagenomics sequencing to profile the human gut microbiome allows for the identification of microbial taxonomic and functional features that are associated with enterolignan production in free-living individuals, although such data are sparse. In addition, it is well elucidated that there are significant between-person variabilities in producing enterolignans upon the consumption of same amount of plant lignans, which might be explained by human gut microbiome [13, 21–23], although no studies have been conducted to evaluate whether the human gut microbiome modulates the association between lignan intake and enterolignan levels, as well as subsequent cardiometabolic risk. To fill these knowledge gaps, we examined the interrelationships between gut microbiome interrogated using shotgun sequencing, plasma enterolactone concentrations, and cardiometabolic risk factors in men participating in the Men’s Lifestyle Validation Study (MLVS). Our primary hypothesis is that there is a consortium of human gut microbes that predict the levels of enterolignans and these microbes modulate the associations between lignan intake and enterolignan levels. In a secondary analysis, we explored whether enterolactone-predicting species also are associated with certain plasma metabolites that might further explain the association between lignan intake and cardiometabolic risk.

## Results

The average intake of total lignans was 3633 ug/day, which were primarily secoisolariciresinol (2957 ug/day), followed by pinoresinol (320 ug/day), lariciresinol (301 ug/day), and matairesinol (32 ug/day). The average plasma enterolactone levels were 29.3 nM (Table 1). The basic characteristics, including intake of energy and alcohol, smoking status, use of antibiotics, and intake of probiotics, were similar across different groups of dietary lignans and plasma enterolactone levels (Table 1).

**Table 1:** Baseline characteristics of 303 men in the Men’s Lifestyle Validation study according to the dietary and plasma lignan levels^a^

	Combination of dietary lignans and plasma enterolactone (EL) levels^3^				Total (*n* = 466)
	Low lignans Low EL (*n* = 141)	High lignans Low EL (*n* = 92)	Low lignans High EL (*n* = 93)	High lignans High EL (*n* = 140)	
**Basic characteristics**^**1**^					
Enterolactone (nM)	9.4 (4.4)	10.0 (4.1)	36.6 (22.9)^a^	56.0 (22.9)^a^	29.3 (34.3)
Lignans (µg/day)	440 (138)	3809 (6996)^a^	465 (133)	8826 (15,996)^a^	3633 (10,209)
Secoisolariciresinol (µg/day)	119 (54)	2561 (6866)	128 (54)	7862 (15,970)^a^	2957 (10,081)
Matairesinol (µg/day)	21 (9)	40 (23)^a^	23 (9)	45 (24)^a^	32 (21)
Lariciresinol (µg/day)	167 (62)	425 (222)^a^	173 (57)	448 (215)^a^	301 (206)
Pinoresinol (µg/day)	134 (72)	560 (777)^a^	140 (69)	479 (495)^a^	320 (458)
Energy intake (kcal/day)	2279 (508)	2400 (423)	2176 (416)	2414 (463)	2164 (664)
Alcohol (g/day)	19.8 (23.4)	20.0 (18.9)	15.1 (16.3)	18.2 (18.4)	18.4 (19.2)
Age (year)	70.9 (4.5)	70.5 (4.0)	71.8 (4.0)	71.0 (4.2)	71.0 (4.2)
Total physical activity (MET-hrs/week)	120 (63)	126 (62)^a^	112 (58)	118 (51)	117 (56.4)
White (%)	95.9	96.6	98.5	97.5	97.0
Smoking (%)	2.2	0.8	2.0	0.0	1.3
Antibiotics use in last 12 months (%)	27.0	26.5	29.5	23.2	26.6
Probiotics use ≥ 1time/week (other than yogurt) in last 2 months (%)	7.0	5.6	5.5	8.5	6.9
**Metabolic risk factors**^**2**^					
Total cholesterol (mg/dL)	177 (31.6)	180 (35.9)	187 (38.8)	186 (33.6)^a^	182 (35.0)
C-reactive protein (mg/dL)	2.3 (3.9)	1.6 (3.1)	1.8 (3.2)	1.2 (2.1)^a^	1.8 (3.3)
HDL-C (mg/dL)	52.5 (14.0)	55.5 (13.3)	57.4 (15.2)	56.7 (13.3)^a^	55.5 (13.9)
Triglycerides (mg/dL)	112 (70.0)	99.4 (45.0)	91.8 (42.3)^a^	93.4 (50.5)^a^	100 (55.9)
HbA1c %	5.8 (0.4)	5.7 (0.3)	5.8 (0.4)	5.7 (0.3)^a^	5.7 (0.4)
Body Mass Index (kg/m^2^)	26.4 (4.0)	25.5 (4.0)	25.1 (3.3)^a^	24.3 (3.1)^a^	25.3 (3.8)
Metabolic score	13.2 (4.0)	12.0 (3.6)	11.7 (3.8)^a^	11.2 (3.6)^a^	12.0 (3.8)

^a^466 measurements from 303 participants with 163 pairs of repeated measurements

^1^High vs low categorization for both lignan intake and enterolactone levels was defined based on the median value of the distribution

^2,3^Values are means (SD) for continuous variables; percentages for categorical variables and are standardized to the age distribution (except age itself) of the study population. Comparing to low dietary low plasma lignan group

^1^*P < 0.05 after adjustment of age distribution

^2^*P < 0.05 after adjustment for repeated measurements (participants ID as random intercept), age, energy intake, alcohol, smoking status, physical activity, and body mass index at age 21

### Gut microbiota taxonomic profile and plasma enterolactone levels

PERMANOVA test based on Bray–Curtis dissimilarities showed that the overall structural variation of the gut microbiome was weakly but significantly associated with plasma enterolactone levels (R^2^ = 0.01, *P* < 0.001,) (Supplementary file Figure S1). After multivariate adjustment of covariates, a total of 18 classified species were significantly associated with enterolactone levels at FDR < 0.05 level (Table 2, Supplementary file Figure S2). For example, the relative abundance of species such as *Coprococcus sp.ART55/1, Faecalibacterium prausnitzii*, *Alistipes shahii, Butyrivibrio crossotus,* and *Methanobrevibacter smithii,* was significantly associated with higher enterolactone levels, and inverse associations were observed for *Bacteroides dorei, B. fragilis, Clostridium bolteae*, *C. leptum*, *C. symbiosum, Lachnospiraceae bacterium.1.4.56FAA*, and *Ruminococcus. sp. 5.1.39BFAA*.

**Table 2:** Multivariable-adjusted associations^1^ between plasma enterolactone and relative abundances of species

**Species**	**N**	**beta coefficient**^2^	**SEM**	***P*-value**	***P***_**FDR**_
*F.coprococcus.sp.art55.1*	148	0.0100	0.0030	0.001	0.02
*F.faecalibacterium.prausnitzii*	901	0.0104	0.0034	0.002	0.03
*B.alistipes.shahii*	712	0.0052	0.0019	0.006	0.045
*F.butyrivibrio.crossotus*	119	0.0069	0.0025	0.007	0.045
*E.methanobrevibacter.smithii*	376	0.0045	0.0017	0.007	0.046
*F.flavonifractor.plautii*	386	-0.0017	0.0005	< 0.0001	0.02
*F.lachnospiraceae.bacterium.1.4.56FAA*	339	-0.0018	0.0006	0.001	0.02
*F.clostridium.bolteae*	453	-0.0046	0.0014	0.001	0.02
*F.clostridium.symbiosum*	317	-0.0017	0.0006	0.002	0.03
*P.escherichia.coli*	395	-0.0087	0.0028	0.002	0.03
*F.blautia.hydrogenotrophica*	260	-0.0015	0.0005	0.004	0.04
*F.veillonella.parvula*	317	-0.0018	0.0006	0.004	0.04
*F.clostridium.leptum*	682	-0.0043	0.0015	0.004	0.04
*F.ruminococcus.sp.5.1.39BFAA*	438	-0.0104	0.0036	0.004	0.04
*F.clostridium.hathewayi*	344	-0.0014	0.0005	0.006	0.045
*B.bacteroides.fragilis*	471	-0.0051	0.0018	0.006	0.045
*B.alistipes.onderdonkii*	750	-0.0076	0.0028	0.006	0.045
*B.bacteroides.dorei*	639	-0.0074	0.0027	0.007	0.045

*Abbreviations: A* Actinobacteria, *B* Bacteroidetes, *F* Firmicutes, *P* Proteobacteria, *E* (Archaea) Euryarchaeota, *N* means the number of samples (out of the 911 samples) with the species detected

^1^Analyzed using MaAsLin 2 adjusted for repeated measurements (participants ID as random intercept), age (year), energy intake (kcal/day), alcohol (g/day), smoking (currently smoking cigarettes or not), physical activity (METs-h/week), using of antibiotics (yes vs. no), consumed any probiotics (yes vs. no), body mass index at age 21 (kg/m^2^) and fecal sample characteristics (6 category groups from hard to soft stool)

^2^beta coefficients: relative abundance of species associated with per standard deviation of enterolactone levels, where enterolactone is batch-corrected log-transformed and in unit of per standard deviation; relative abundance of species was standardized and normalized via arc-sin square root transformation

*P*_*FDR*_* P* values after false discovery rate (FDR) correction following the Benjamini–Hochberg method

### Interactions between microbiome and lignan intake on plasma enterolactone levels

**Fig. 1:**
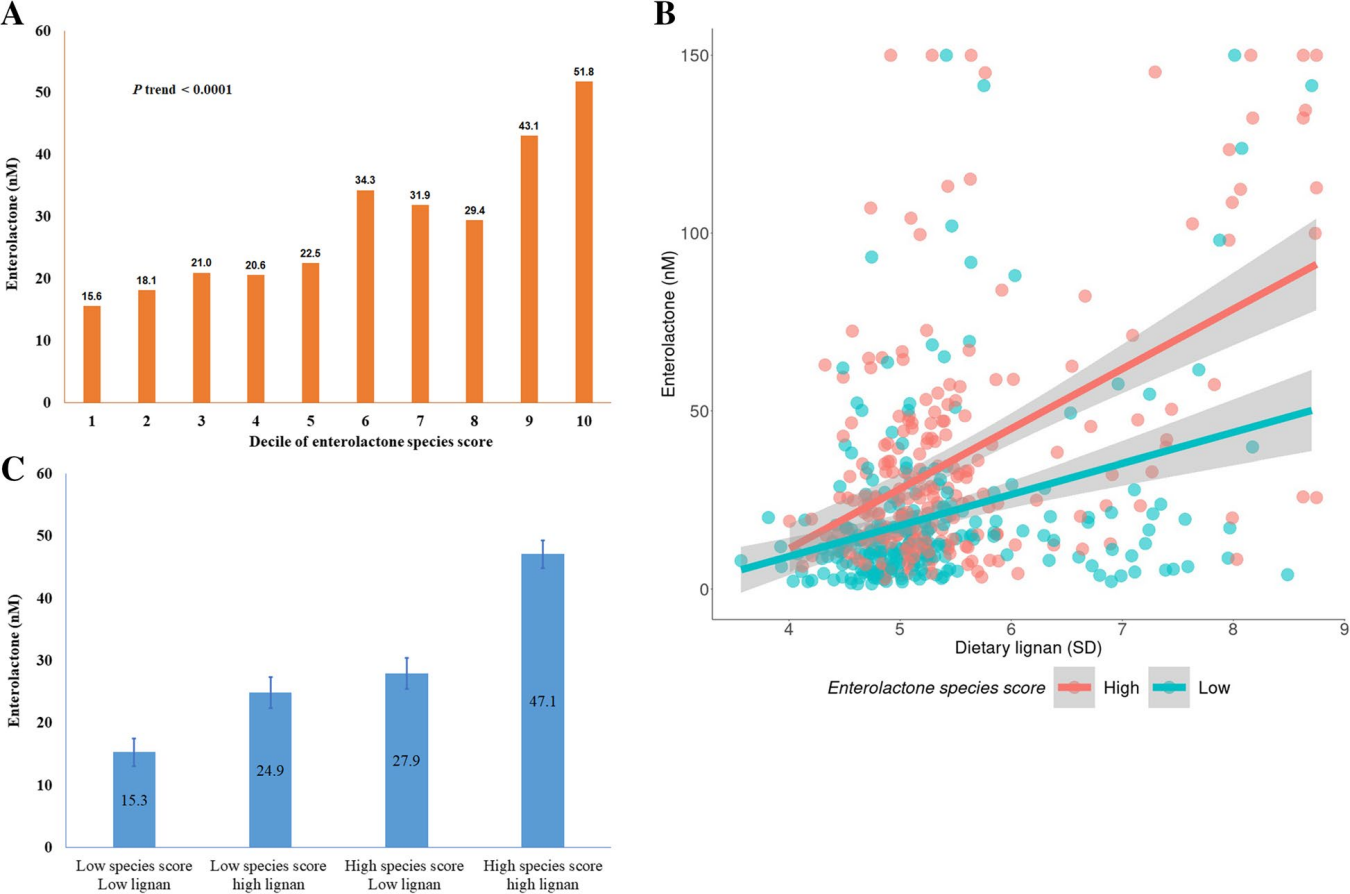
Plasma enterolactone according to dietary lignans and species score. (**A**) Enterolactone increased with deciles of enterolactone species score (P trend < 0.0001); (**B**) Association between dietary lignan and enterolactone stratified by enterolactone species score (P for interaction<0.001; Among group with low species score: Beta: 4.0; SE: 1.7nM per SD lignan, P trend <0.0001; Among group with high species score: Beta: 20.7; SE: 2.3nM per SD lignan, P trend <0.0001); (**C**) Enterolactone according to joint classification of dietary lignan and enterolactone species score. ^*^Generalized linear mixed-effects regressions adjusted for repeated measurements (participant ID as random intercept), age (year), energy intake (kcal/day), alcohol (g/day), smoking (currently smoking cigarettes or not), physical activity (METs-h/week), using of antibiotics (yes vs. no), consumed any probiotics (yes vs. no), body mass index at age 21 (kg/m^2^) and fecal sample characteristics (6 category groups from hard to soft stool)

To summarize the microbial composition that was significantly associated with enterolactone levels, we built a rank-based, un-weighted species score based on the relative abundances or absence/presence of all 18 species. The multivariate-adjusted mean value of enterolactone was 15.6 nM (SEE: 5.0) among participants in the lowest decile of species score and 51.8 nM (SEE: 5.4) among participants in the top decile (*P* for trend < 0.0001, Fig. 1A). Lignan intake significantly interacted with the species score on plasma levels of enterolignans: dietary lignan (per SD) was associated with 4.0 nM higher enterolactone levels among participants with a lower species score, however the increment was 20.7 nM per SD lignan intake among participants with a higher species score (*P* for interaction < 0.001, Fig. 1B). The multivariate-adjusted means of enterolactone levels were 15.3 nM, 24.7 nM, 27.9 nM, and 47.1 nM, respectively, among participants with low lignan-low species score, high lignan-low species score, low lignan-high species, and participants with high lignan-high species score (Fig. 1C).

### Lignan intake, plasma enterolactone levels, and metabolic risk

**Fig. 2:**
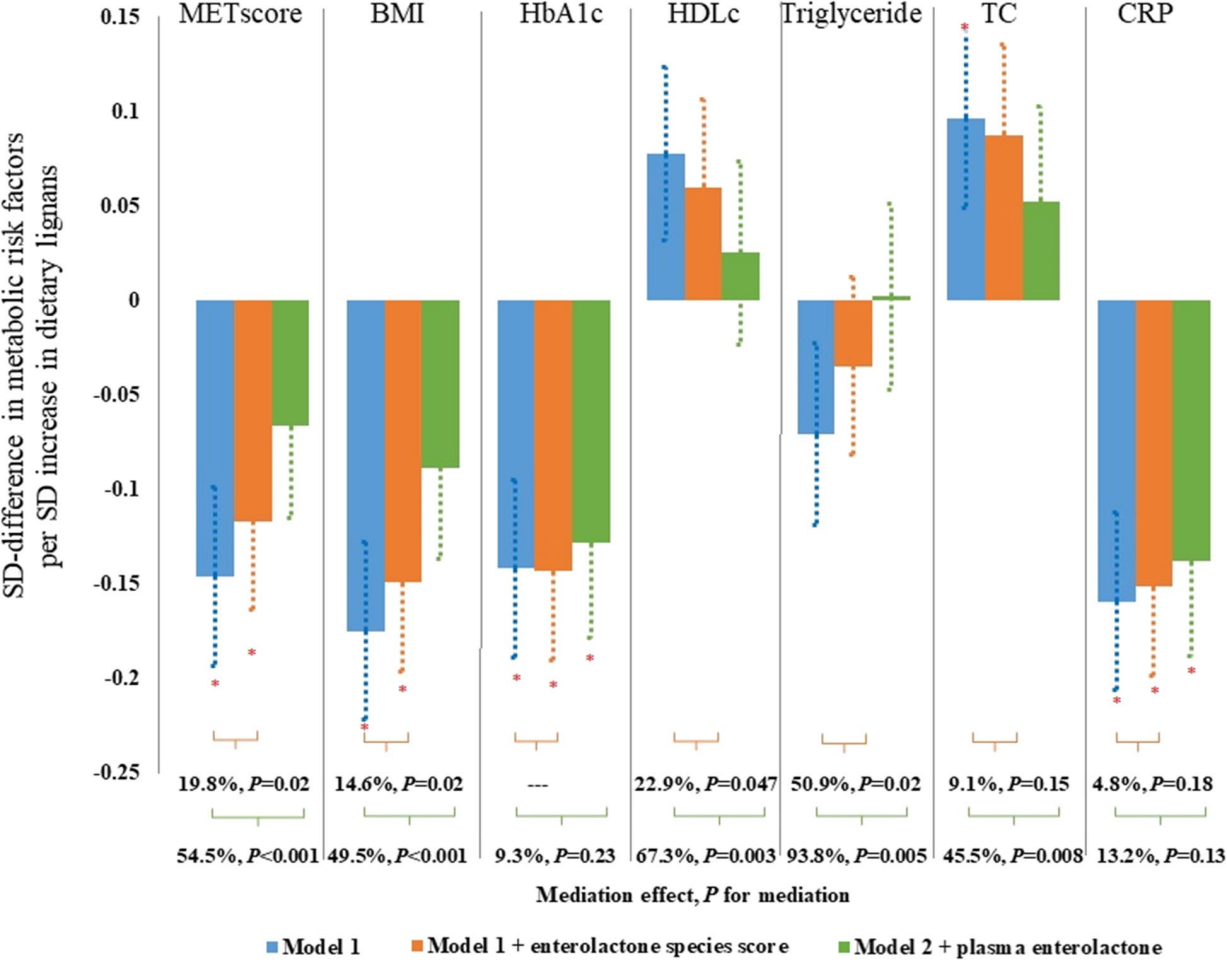
Association between dietary lignans and metabolic risk factors and potential mediation effect by enterolactone and enterolactone-predicting species score. ^*^P < 0.05 for Beta (SEE) of the change of metabolic risk factors associated with per standard deviation (SD) changes of log-transferred dietary lignans; Model 1: Generalized linear mixed-effects regressions adjusted for repeated measurements (participant ID as random intercept), age (year), energy intake (kcal/day), alcohol (g/day), smoking (currently smoking cigarettes or not), physical activity (METs-h/week), using of antibiotics (yes vs. no), consumed any probiotics (yes vs. no), body mass index at age 21 (kg/m^2^) and fecal sample characteristics (6 category groups from hard to soft stool). Model 2: Model 1 further adjusted for enterolactone related species score. Model 3: Model 2 further adjusted for plasma enterolactone level. Mediation effect: proportion of dietary lignan effects potentially mediated by enterolactone species score (orange) or plasma enterolactone (green). P for interactions between lignans and enterolactone species score were 0.35 for metabolic score, 0.66 for BMI, 0.38 for HbA1c, 0.46 for HDL_C, 0.33 for TG, 0.49 for TC and 0.33 for CRP; P for interactions between lignans and enterolactone were 0.58 for metabolic score, 0.81 for BMI, 0.06 for HbA1c, 0.95 for HDL_C, 0.36 for TG, 0.33 for TC and 0.42 for CRP

Dietary lignan was significantly associated with a lower level of BMI, HbA1c, CRP, and the overall metabolic risk score, and these associations were mainly mediated by both the species score and plasma enterolactone levels (Fig. 2). For example, lignan intake (per SD) was inversely associated with the metabolic risk score (β = -0.15 SD, SEE: 0.05, *P*_trend_ = 0.003), 19.8% (95% CI: 7.3%, 43.6%) of which was explained by the species score and 54.5% (95% CI: 21.8%, 83.7%) jointly by the species score and plasma enterolactone levels. We did not detect any significant interaction between lignan intake and the species score on metabolic risk score or individual metabolic risk factors (Fig. 2).

### Microbial compositions potentials of super pathways and plasma enterolactone levels

Overall, of the 110 super pathways considered in the analyses after filtering, the DNA abundance of 55 pathways was significantly associated with plasma enterolactone levels (FDR < 0.05), including 13 enriched and 42 depleted super pathways (Supplementary file Table S1), such as enriched branched amino acid biosynthesis, 5-aminoimidazole ribonucleotide biosynthesis, L-isoleucine biosynthesis I, L-serine, glycine biosynthesis I, hexuronide and hexuronate degradation and β-D-glucuronide and D-glucuronate degradation. We further examined the functional profile at the enzyme level, enterolactone was significantly associated with the relative abundance of 229 enzymes (FDR < 0.05, Supplemental Table S2), including 133 depleted enzymes, such as endo-1,3(4)-beta-glucanase (EC 3.2.1.6), and n-acetylgalactosamine-4-sulfatase (EC 3.1.6.12), and 96 enriched enzymes, such as glycogen phosphorylase (EC 2.4.1.1), 1,2-diacylglycerol 3-glucosyltransferase (EC 2.4.1.157), and glucosylceramidase (EC 3.2.1.45).

### Plasma metabolites, microbiome, and metabolic risk

**Fig. 3:**
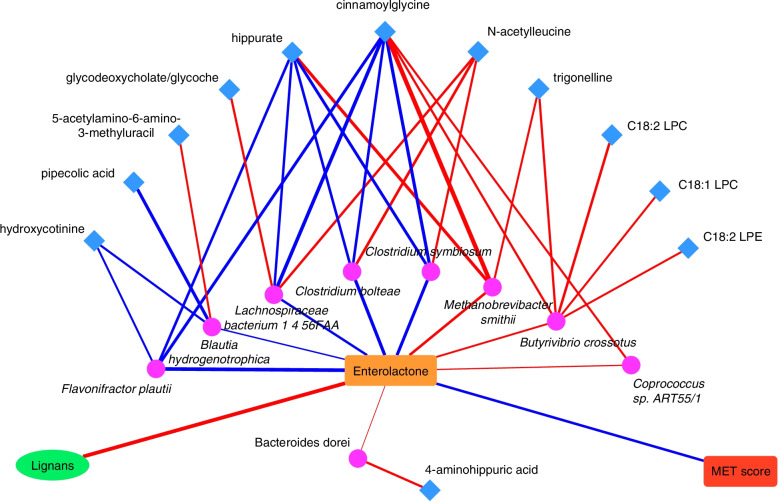
Network between enterolactone-predicting species and plasma metabolites. (Lines indicate the correlations between two components: red are positive correlations and blue are inverse correlations while the size of lines indicates the relative strength of the correlation coefficients; pink circles represent species and blue squares represent metabolites)

Of the 190 metabolites included in the analysis, 12 metabolites, including cinnamoylglycine, hippurate, pipecolic acid, C18:1 lysophosphatidylcholine (LPC), C18:2 LPC, C18:2 lysophosphatidylethanolamine, 2-aminohippuric acid, 5-acetylamino-6-amino-3-methyluracil, hydroxycotinine, N-acetylleucine, trigonelline and glycodeoxycholate/glycoche, exhibited significant associations with the relative abundance of several enterolactone-predicting species, such as *M. smithii*; *B. crossotus, C. symbiosum*, and *L. bacterium 1.4.56FAA* (Fig. 3, Supplementary file Table S3). The 12 metabolites, together with plasma enterolactone and the stool microbiome species score, jointly explained 79.8% (95%CI: 17.7%, 98.6%; *P* = 0.002) of the association between lignan intake and the metabolic risk score.

## Discussion

In the present study, we aimed to examine the role of the microbiome in the inter-connections between lignan intake, plasma enterolactone concentrations, and metabolic health in free-living healthy men. We identified multiple gut microbiota species and multiple microbial functional metabolic pathways that were significantly associated with plasma enterolactone concentrations. The association between dietary lignans and plasma enterolactone concentrations was significantly stronger among participants with a microbiota taxonomic profile that predicted higher enterolactone concentrations. In addition, the favorable association of dietary lignans with metabolic risk was partially mediated by enterolactone levels and the microbiome profile jointly. Lastly, the species that predicted enterolactone concentrations also predicted levels of certain plasma metabolites, such as cinnamoylglycine, glycine, and C18:2 LPC, which further explained the association between lignan intake and metabolic risk.

To date, no single bacteria species or strains that could independently convert plant lignans to enterolignans have been identified [24]. Results from recent studies [16, 25] suggest that the metabolism of lignans involves phylogenetically diverse bacteria and multiple species from distinct bacterial phyla to produce bioactive enterolignans. Conventional culture-based studies are currently the main source of our knowledge on gut bacteria that may mediate these reactions. For example, Clavel et al. [13–17] demonstrated that *C. saccharogumia*, *C. cocleatum*, *C. ramosum*, *B. fragilis*, *B. ovatus*, and *B. distasonis* were able to deglycosylate plant lignans, whereas *Butyribacterium methylotrophicum*, *Eubacterium callanderi*, *E. limosum*, *E. callanderi, Blautia product*, *and Butyribacterium methylotrophicum* were capable of demethylation. Other bacteria, such as *R. bromii*, *R. lactaris*, *Bifidobacterium strains*, *E. lenta*, *Enteroccus faecalis*, *C. scindens*, *Lactonifactor longoviformis*, *Peptostreptococcus productus*, *C. coccoides, Ruminococcus* strains, *Eubacterium* strains, and *B. pseudocatenulatum*, can mediate these reactions as well [16–20, 26–30]. However, it is unknown whether these findings can be entirely generalizable to epidemiological study settings.

In the current study among free-living male adults, we replicated certain previously culture-based studies identified species and genera, which are known to be involved in the conversion of plant lignans to enterolactone [16]. Specifically, *B. ovatus* is known to deglycosylate plant lignans [15–20]. *M. smithii*, the dominant archaea in the human intestinal ecosystem, can enhance the bacterial digestion of dietary polysaccharides and is critical for the syntrophic hydrogen metabolism, which is important to enterolactone production [31, 32]. Meanwhile, through interrogating the metagenomic profiling data, we also identified a wide range of novel species that were significantly associated with enterolactone levels. In particular, *F. prausnitzii*, one of the most abundant bacterial species in human gut, is well known as an anaerobic fiber metabolizer, and for its implication as a biomarker for diagnostics and prognostics of gastrointestinal diseases, such as ulcerative colitis and Crohn’s disease [24, 33, 34]. Our findings echo the notion that the enterolignan production involves a consortium of phylogenetically-distinct bacteria and also highlight the potentials of using modern metagenomic profiling to aid in the discovery of novel taxonomic features that are associated with biomarkers or disease outcomes. Nonetheless, our findings warrant further investigations and replications in future studies, especially in different populations that include females and broader race/ethnicity mix.

It is well-documented that the production of enterolignans from lignan intake is highly individualized, and such individualized responses might be largely ascribed to the microbial composition [21, 35]. Our study highlights, for the first time, that the gut microbial species that predict plasma enterolactone levels also significantly modulate the relationship between lignan intake and plasma enterolactone levels. Specifically, we observed a much stronger association between lignan intake and enterolactone levels when the microbiota was enriched with species that predicted higher enterolactone levels and/or depleted with species that predicted lower levels. This finding has a clear implication for developing a precision nutrition approach to enhancing the production of bioactive microbiota metabolites, such as enterolignans, through modulating the microbial composition, which may subsequently lead to improved metabolic health.

Of note, the greater production of enterolactone did not translate to a stronger inverse association between lignan intake and metabolic risk. Nonetheless, in the current study, both gut microbial profiles and plasma enterolactone levels accounted for significant proportion of the inverse association between lignan intake and metabolic risk. Existing literature also suggests potential beneficial effects of higher enterolactone levels or lignan intake on cardiometabolic conditions, including coronary heart disease and type 2 diabetes [36–40]. The mechanisms underlying lignans and enterolignans’ health benefits may be through inhibiting lipid peroxidation [41–43], reducing oxygen species production [44, 45], inducing gene expression of antioxidant enzymes [46], and reducing vitamin E catabolism [47]. The structural similarity of enterolactone to 17β-estradiol allows enterolignans to bind to estrogen receptor alpha (ERα) and exert weak estrogenic or anti-estrogenic effects [48]. Lignans also increase the levels of sex-hormone-binding protein [49, 50], which leads to reduced free estradiol, improved insulin resistance [51, 52], and a lower diabetes risk. Lignans may also improve insulin resistance through inhibiting pancreatic α-amylase [53] and decreasing inflammation [41].

It is not surprising that we found a few pathways of housekeeping genes, such as amino acid synthesis and nucleotide synthesis pathways, that were associated with enterolactone levels given that multiple species were significantly associated with enterolactone levels. Interestingly, several of the enterolactone-predicting species were also associated with plasma levels of other metabolites, such as hippurate and cinnamoylglycine, both of which are microbiota-dependent glycine-conjugated metabolites [54, 55]. Our finding is consistent with a previous cross-sectional analysis that showed a significant association between levels of enterolactone and hippuric acid in plasma [56]. In addition, another cross-sectional analysis in the TwinsUK cohort showed significant inter-relationships between diet, *F. prausnitzii* abundance, plasma hippurate levels, and metabolic syndrome [57]. A more recent analysis in the same cohort showed that plasma levels of cinnamoylglycine was positively associated with the microbiome α diversity and inversely associated with glucose level and obesity [58]. Studies reported significant associations between total or specific LPC concentrations with lower metabolic and diabetes risk [59, 60], though the role of LPCs in metabolic pathophysiology is not completely understood and the role of microbiota is not clear. Enzyme level analysis provided further supports of the enterolactone related species at function level, such as glucosyltransferases that played an important role in the lignan metabolism [61, 62], was significantly associated with enterolactone level in our study. Overall, this exploratory analysis demonstrated novel, inter-relationships between microbiome and plasma metabolites that might be relevant to enterolactone metabolism, although longitudinal studies are needed to establish temporal relationships with metabolic risk.

The current analysis leveraged repeated assessments of diet, gut microbiome, and metabolic risk markers, which reduces error due to within-person variability. The primary limitation of this study was the cross-sectional design and observational nature of the data, which limited causal interpretations of the findings. Our findings shall be replicated in future epidemiological studies and culture-based in vitro experiments. In addition, since our study participants were older male health professionals who are mostly white, whether the findings can be generalized to women, younger population, individuals of other racial/ethnical groups, or populations with different demographic characteristics warrant further investigations.

## Conclusion

In conclusion, in a group of healthy men we identified multiple gut microbiota species that were significantly associated with plasma enterolactone levels. A microbial profile defined by the species was significantly associated with plasma enterolactone concentrations and also significantly strengthened the relationship between plant lignan intake and plasma enterolactone levels. Both the enterolactone and the microbial profile that predicted higher enterolactone levels mediated association between higher lignan intake and lower metabolic risk. Plasma metabolites that were associated with the enterolactone-predicting species further explained the association of lignan intake with metabolic risk. These findings underscore a pivotal role of microbiome in modulating and mediating inter-relationships between diet, gut microbiota-derived metabolites, and metabolic risk.

## Methods

### Study population

The MLVS is a sub-study conducted in 2012–2013 within the Health Professionals Follow-up Study (HPFS) cohort with a goal of examining the validity of self-reported dietary and lifestyle assessments The HPFS was established in 1986, when 51,529 male US health professionals aged 40–75 years completed a mailed questionnaire about their medical history and lifestyle at baseline, and follow-up questionnaires have been administered to assess and update lifestyle, diet, and medical history.

Briefly, HPFS participants who provided blood samples during 1994–1995, participated in the 2010 HPFS follow-up survey with a valid food frequency questionnaire (FFQ), and were free of cardiovascular disease, cancer, and major neurological diseases, were eligible to participate in the MLVS. A total of 648 eligible HPFS men participated in the MLVS and accomplished multiple examinations, including dietary assessments, collection of blood and fecal samples, and assessments of lifestyle factors within a year. In the MLVS and HPFS, demographics, anthropometry, diet, lifestyle, medical history, and other data were collected/updated during follow-up. Covariates, including total energy intake, physical activity, smoking, alcohol consumption, BMI at age 21, and other variables, were derived in the MLVS and HPFS. The current analysis was restricted to 303 MLVS participants who provided fecal samples, completed 7-day diet records (7DDRs), provided blood sample for the measurement of cardiometabolic biomarkers, and had valid enterolactone measurements (Supplemental file Figure S3).

The study protocol was approved by the Harvard T.H. Chan School of Public Health Institutional Review Board, and informed consent was obtained from all participants.

### Fecal sample collection

A total of 308 MLVS participants provided up to two pairs of stool samples. These participants were on average 71 years old (range: 65–82) at fecal sample collections. In addition, two blood samples were collected 6 months apart roughly at the same time of the fecal sample collections. Fecal sample collections were self-administered through deposition in a commode specimen collection system (Fisher Scientific). Participants were requested to provide two pairs of fecal samples (6 months apart) from two consecutive bowel movements and instructed to put a small sample of stool in collection tubes with RNAlater preservative. The time interval between two consecutive fecal sample collections was 24–72 h. Participants then shipped the tubes to the cohort biorepository within 24 h via prepaid overnight FedEx. A questionnaire was administered to collect information at fecal sample collections, such as the Bristol Stool Chart, use of antibiotic medications, age, and intake of any probiotics (except yogurt) in the preceding 2 months.

### Dietary assessments

In the MLVS, diet was assessed twice via 7DDRs. Briefly, participants received a food scale (Escali Corporation, Burnsville, Minnesota) and ruler, an instructional DVD, and instructions via telephone. Participants measured and reported weights (gram) of foods before and after eating so actual intake could be computed. The participants also provided recipes of all home-prepared foods, including the number of servings in each recipe and the portion of the recipe they consumed. The food intake data were then combined with the Nutrition Data System for Research 2011 database to calculate the dietary intakes of total lignans as well as 4 individual lignans, including secoisolariciresinol, matairesinol, lariciresinol, and pinoresinol.

### Taxonomic and functional profiling of metagenomic samples

Detailed information of sample collection and immediate ex situ conservation of metagenomic data, laboratory handling, and paired-end (100 × 100 nucleotides (nt)) shotgun sequencing of DNA can be found in previous publications of the MLVS [63, 64]. Standard protocols were applied to extract DNA from fecal samples. The Nextera XT DNA Library Preparation Kit was subsequently used to prepare DNA samples for sequencing. DNA was then sequenced to a target depth of 1-2Gnt each. The bioBakery workflow was used to generate taxonomic and functional profiles [65]. Briefly, quality controls included depletion of duplicate reads using KneadData (http://huttenhower.sph.harvard.edu/kneaddata), the removal of human sequences, functional profiling by HUMAnN2 [67], and taxonomic profiling by MetaPhlAn2 [66]. DNA reads were assigned to UniRef90 gene families by HUMAnN2; then the characterized gene families were assigned to MetaCyc pathways [68], as described in detail elsewhere [64]. The current analysis included 911 metagenomes from the 303 MLVS participants. We further filtered out taxonomic features with a relative abundance less than 10^−4^ in greater than 10% of all samples. We filtered all gene families with a relative abundance less than 10^−5^ in greater than 10% of all samples.

### Assessment of enterolactone and metabolic risk factors

For measurements of enterolactone and biomarkers, repeated fasting blood samples were collected 6 months apart through venipuncture into sodium heparin tubes, shipped by overnight mail with an ice pack, and then were processed to separate the plasma upon arrival at the cohort biorepository. Electrospray ionization orbitrap liquid chromatography mass spectrometry (ESI-LCMS; model Q-Exactive, Thermo Scientific Inc., Waltham, MA) in negative mode148 was used to measure enterolactone concentrations in plasma [69].

Detailed measurement and assessment of metabolic risk score is described previously [70]. In brief, plasma levels of high-density lipoprotein (HDL)-cholesterol and triglycerides were assayed using enzymatic methods. Hemoglobin A1c (HbA1c) levels were measured using the turbidimetric immunoinhibition (Roche Diagnostics). Blinded quality control samples (10%) were interspersed among participants’ samples. Intra-assay coefficients of variation (CVs) were estimated to be < 7% for all plasma markers, except enterolactone for which the CV was 17%. Between-batch variations of enterolactone measurements were corrected using a standardized batch analytic method [70]

Four of the five individual components of metabolic syndrome [71], including triglycerides, HDL-C, body mass index (BMI), and HbA1c, were used to derive a metabolic risk score in the current analysis. Blood pressure or fasting glucose assessments were not measured in the MLVS. We first assigned a score of 1–5 to quintiles of each of the 4 factors, with 1 assigned to the lowest quintile and 5 to the highest quintile for triglycerides, BMI and HbA1c. The scoring algorithm was reversed for HDL-C that 1 was assigned for the highest quintile and 5 for the lowest quintile. The metabolic risk score was the sum of the 4 components scores with a theoretical range of 5 (lowest possible score, healthy) to 20 (highest possible score, unhealthy).

### Plasma metabolomics profiling

Plasma metabolome was profiled using high-throughput liquid chromatography-mass spectrometry (LC–MS) techniques at the Broad Institute of MIT and Harvard [72]. We used hydrophilic interaction liquid chromatography with positive ionization mode mass spectrometry detection (HILIC-pos) to separate polar metabolites. Raw data were processed using TraceFinder software (Thermo Fisher Scientific) and Progenesis QI (Nonlinear Dynamics, Newcastle upon Tyne, UK). Known metabolite identities were confirmed using authentic reference standards. A total of 201 known metabolites were profiled. Metabolites with missing values in more than 25% of the participant samples were excluded from the analysis (*n* = 11), leaving 190 metabolites. The rest of metabolites that were below level of detection were replaced using 1/2 minimum value of detected metabolites.

### Statistical analysis

The analyses involving the relative abundance of microbial species and super pathways (DNA) were based on the 911 metagenome assessments. We matched each of the microbiome measurements to biomarkers, diet, and other variables collected at the time closest to the time of fecal sample collection. For example, the 1st and 2nd metagenomes (i.e., the first pair of fecal samples) were each matched to biomarkers measured at the first blood collection, and average intake of lignans from the first set of 7DDRs. The same strategy was used to match the second pair of fecal metagenomes with corresponding diet/biomarker measurements.

We applied normalized via arc-sin square root transformation for all species/pathway data and then used MaAslin 2 (https://huttenhower.sph.harvard.edu/maaslin2) to examine the associations between the enterolactone concentrations and the relative abundance of taxonomy and pathways. Within-person correlations between the up to four data points per person was accounted with a random effect built in MaAslin 2. We used Graphical Phylogenetic Analysis (GraPhlAn) (https://huttenhower.sph.harvard.edu/graphlan) to visualize the results of taxonomies. Covariates considered in multivariate analyses included age, physical activity, BMI at age 21, alcohol consumption, smoking, Bristol Stool Chart categories, use of antibiotics in preceding year, intake of any probiotics (except yogurt) in the preceding 2 months, and total energy intake. *P* values below 0.05 after false discovery rate (FDR) correction following the Benjamini–Hochberg method were considered statistically significant.

To further alleviate multiple comparison concerns, we developed an un-weighted score [73] to summarize the abundance of species that were significantly associated with enterolactone levels in the primary analyses at FDR < 0.05. To derive the species score, for species that were detected in ≥ 455 (of the 911) samples, we categorized them as being either “high” or “low” by the median relative abundant value. For species that were detected in < 455 (of the 911) samples, the binary categories were based on the presence or absence of the species. We then assigned 1 for higher abundance or presence of species that were positively associated with enterolactone or 0 otherwise, and we reversed the score for species that were inversely associated with enterolactone levels. We then summed the scores across all species to derive an overall species score.

To examine potential interactions between lignan intake and the gut microbiome on plasma levels of enterolactone, we defined the low and high species score using the median value as the cutoff point. The interaction between dietary lignans and species score on enterolactone was tested by further including the interaction term of dietary lignans and species score in a multivariate adjusted linear mixed model that included both dietary lignan intake and species score as well as the covariates listed above. To examine the extent to which plasma enterolactone levels and the species score explained the association between dietary lignans and the metabolic risk score, we estimated the magnitude of change in the regression coefficient for dietary lignans with and without adjustment for species score and/or plasma enterolactone level. The percentage of association between dietary lignans and metabolic risk that was explained by the species score or enterolactone was computed as follows: (1 –(β_1_ / β_2_)) X 100% using the %MEDIATE SAS macro (publicly available at www.hsph.harvard.edu/faculty/spiegelman/mediate.html), where β_2_ was the regression coefficient of dietary lignans without adjustment of the species score and enterolactones and β_1_ was the coefficient with such an adjustment. Similarly, in these analyses the averaged species score from the first and second metagenomic assessments were aligned with the first dietary and metabolic risk score assessments, while the averaged species score from the third and fourth metagenomic assessments were matched to the second dietary and metabolic risk score measurements.

In the secondary analysis that explored the role of other plasma metabolites in the inter-relationship between lignan intake, microbiome, and metabolic risk, all plasma metabolites were normalized after log-transformation. This analysis was focused on the metabolites that were associated with the species that significantly predicted enterolactone levels. Partial Spearman correlation coefficients adjusted for the same covariates listed above were used to evaluate the associations between the species and the metabolites. The potential mediational role of the metabolites in the association between dietary lignans and metabolic risk was examined. The correlations were visualized using Cytoscape (https://cytoscape.org/). The significant level was set at *P* values below 0.05 after false discovery rate (FDR) correction, which corresponds to partial Spearman correlation coefficients above 0.2 or below -0.2.

## Supplementary Information

**Additional file 1:** **Supplemental Table S2.** list of enzymes that significantly associated with enterolactone concentration.

**Additional file 2:** **Supplementary Figure S1.** Association between enterolactone and microbial community (PERMANOVA with Bray-Curtis dissimilarities: R2=0.01044, P<0.001). **Supplemental Figure S2.** Taxonomic tree with highlighted species that significantly associated with enterolactone concentrations. **Supplemental Figure S3.** Flowchart of participant enrollment. **Supplementary Table S1.** Multivariable-adjusted associations between plasma enterolactone and relative abundances of genetic predisposition of super pathways*. **Supplementary Table S3.** Plasma metabolites significantly associated with enterolactone-predicting species.

## Data Availability

Sequence data have been deposited in the Sequence Read Archive under BioProject accession PRJNA354235. The datasets used and analyzed during the current study are available from the corresponding author on reasonable request with standard internal approval procedure. Access is restricted due to participant confidentiality and privacy concerns. Further information including the procedures to obtain and access data from the Health Professionals Follow-up Study is described at https://sites.sph.harvard.edu/hpfs/resources-for-hpfs-investigators/.
