# Supplementary material for: Dietary lignans, plasma enterolactone levels, and metabolic risk in men: exploring the role of the gut microbiome
Source: BMC Microbiol. 2022 Mar 29;22:82. doi: 10.1186/s12866-022-02495-0 (PMC8966171; doi:10.1186/s12866-022-02495-0)
Supplement: Supplementary file 2 — Additional file 2: Supplementary Figure S1. Association between enterolactone and microbial community (PERMANOVA with Bray-Curtis dissimilarities: R2=0.01044, P<0.001). Supplemental Figure S2. Taxonomic tree with highlighted species that significantly associated with enterolactone concentrations. Supplemental Figure S3. Flowchart of participant enrollment. Supplementary Table S1. Multivariable-adjusted associations between plasma enterolactone and relative abundances of genetic predisposition of super pathways*. Supplementary Table S3. Plasma metabolites significantly associated with enterolactone-predicting species. [file 12866_2022_2495_MOESM2_ESM.docx]

**Supplementary Materials for Li et al. Dietary lignans, plasma enterolactone levels, and metabolic risk: exploring the role of the gut microbiome**

**Supplementary Figure S1 Association between enterolactone and microbial community**

**(PERMANOVA with Bray-Curtis dissimilarities: R^2^=0.01044, *P*<0.001)**

**
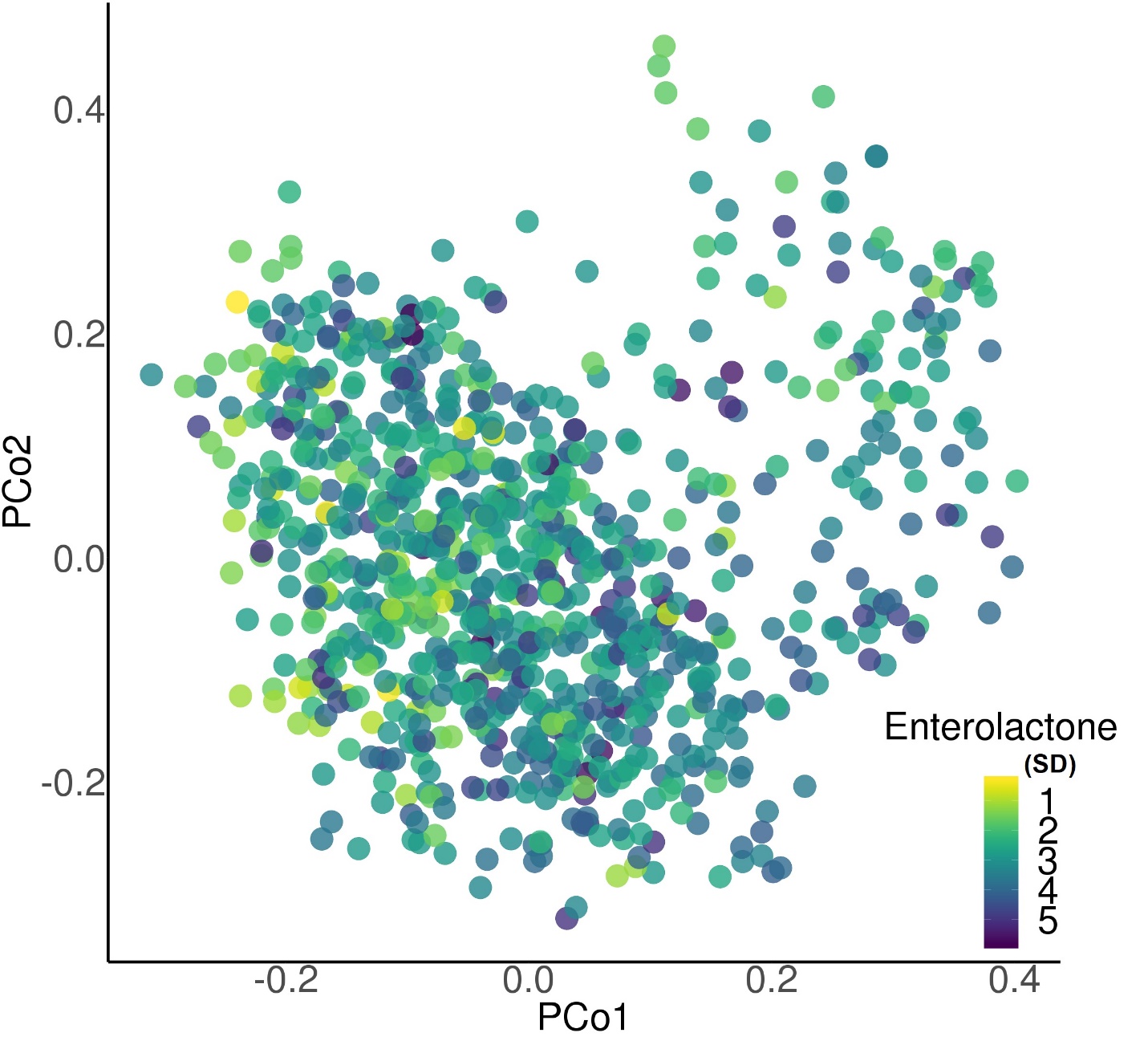
**

**Supplemental Figure S2: Taxonomic tree with highlighted species that significantly associated with enterolactone concentrations**

**
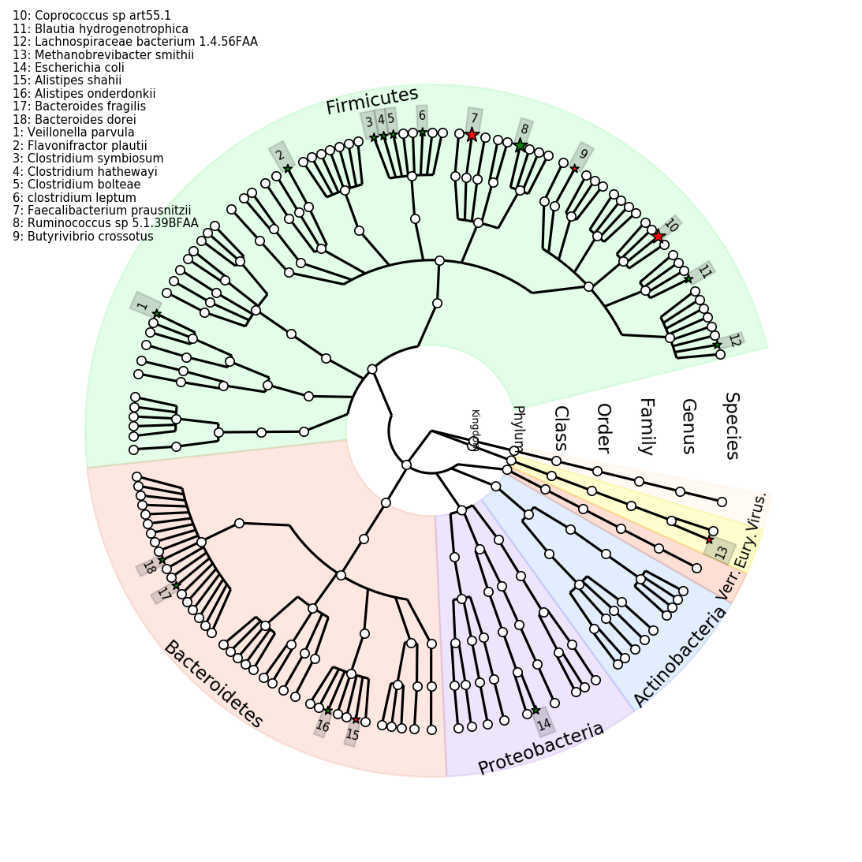
**

(Species were filled with red if increasing enterolactone was associated with a higher abundance; filled with **green** if increasing enterolactone was associated with a decreasing abundances of species; empty circles meant non-significant associations at FDR<0.05 level; stars mean the FDR<0.05. Generalized linear mixed-effects regressions implemented in MaAsLin2 were adjusted for repeated measurements (participants ID as random intercept), age, energy intake, alcohol, smoking, physical activity, using of antibiotics, consumed any probiotics and fecal sample characteristics.)

**Supplemental Figure S3: Flowchart of participant enrollment.**

**
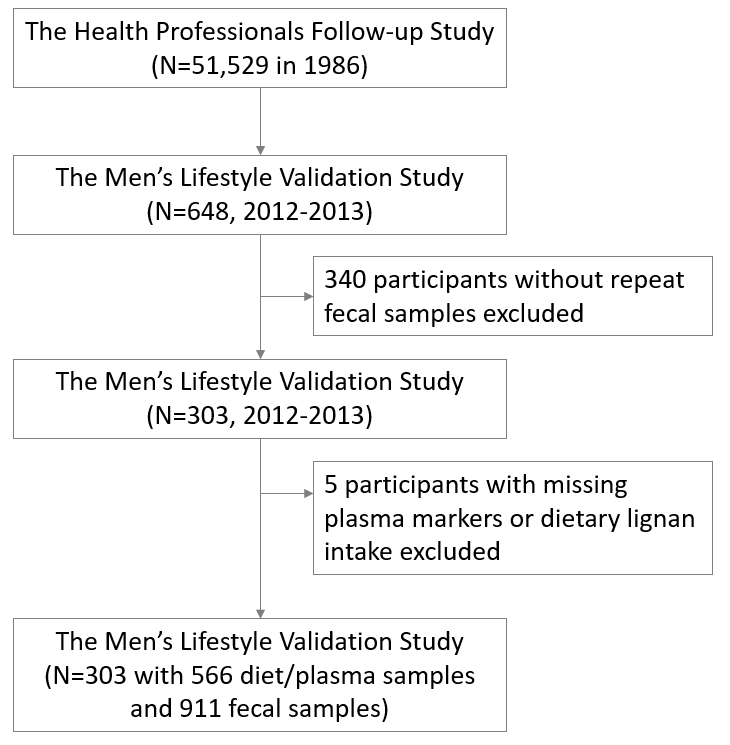
**

**Supplementary Table S1 Multivariable-adjusted associations between plasma enterolactone and relative abundances of genetic predisposition of super pathways^*^**

| **Super Pathways** | **N** | **beta coefficient^2^** | **SEM** | ***P*-value** | ***P*_FDR_** |
| --- | --- | --- | --- | --- | --- |
| Branched amino acid biosynthesis | 909 | 0.0068 | 0.0012 | <.0001 | <.0001 |
| 5-aminoimidazole ribonucleotide biosynthesis | 911 | 0.0050 | 0.0013 | 0.0001 | 0.002 |
| L-isoleucine biosynthesis I | 910 | 0.0032 | 0.0008 | 0.0001 | 0.002 |
| L-serine and glycine biosynthesis I | 907 | 0.0040 | 0.0012 | 0.0006 | 0.003 |
| Purine nucleotides de novo biosynthesis I | 903 | 0.0030 | 0.0011 | 0.005 | 0.01 |
| Aromatic amino acid biosynthesis | 910 | 0.0035 | 0.0013 | 0.008 | 0.02 |
| L-lysine, l-threonine and l-methionine biosynthesis II | 911 | 0.0029 | 0.0011 | 0.008 | 0.02 |
| Hexuronide and hexuronate degradation | 906 | 0.0025 | 0.0010 | 0.009 | 0.02 |
| Thiamin diphosphate biosynthesis III (eukaryotes) | 908 | 0.0023 | 0.0009 | 0.010 | 0.02 |
| Polyamine biosynthesis I | 893 | 0.0023 | 0.0009 | 0.01 | 0.03 |
| Purine nucleotides de novo biosynthesis II | 900 | 0.0022 | 0.0009 | 0.02 | 0.04 |
| ß-D-glucuronide and D-glucuronate degradation | 907 | 0.0022 | 0.0010 | 0.03 | 0.0478 |
| Histidine, purine, and pyrimidine biosynthesis | 887 | 0.0024 | 0.0011 | 0.03 | 0.0496 |
| Purine nucleotide salvage | 878 | -0.0064 | 0.0014 | <.0001 | 0.0003 |
| Unsaturated fatty acids biosynthesis (e. coli) | 684 | -0.0049 | 0.0012 | <.0001 | 0.0009 |
| Mycolate biosynthesis | 684 | -0.0051 | 0.0012 | <.0001 | 0.001 |
| Purine deoxyribonucleosides degradation | 896 | -0.0040 | 0.0010 | 0.0001 | 0.002 |
| L-phenylalanine biosynthesis | 501 | -0.0054 | 0.0014 | 0.0001 | 0.002 |
| Heme biosynthesis from uroporphyrinogen-iii | 282 | -0.0020 | 0.0005 | 0.0003 | 0.002 |
| Demethylmenaquinol-6 biosynthesis I | 190 | -0.0028 | 0.0008 | 0.0003 | 0.002 |
| Demethylmenaquinol-9 biosynthesis | 190 | -0.0028 | 0.0008 | 0.0003 | 0.002 |
| Menaquinol-9 biosynthesis | 190 | -0.0033 | 0.0009 | 0.0003 | 0.002 |
| Menaquinol-6 biosynthesis I | 190 | -0.0033 | 0.0009 | 0.0003 | 0.002 |
| Menaquinol-10 biosynthesis | 190 | -0.0033 | 0.0009 | 0.0003 | 0.002 |
| Allantoin degradation In plants | 182 | -0.0010 | 0.0003 | 0.0006 | 0.003 |
| (Kdo)2-lipid A biosynthesis | 203 | -0.0015 | 0.0004 | 0.0006 | 0.003 |
| Methylglyoxal degradation | 207 | -0.0016 | 0.0005 | 0.0006 | 0.003 |
| Cytosolic glycolysis (plants), pyruvate dehydrogenase and TCA cycle | 239 | -0.0022 | 0.0007 | 0.001 | 0.006 |
| Glyoxylate cycle and fatty acid degradation | 276 | -0.0022 | 0.0007 | 0.002 | 0.009 |
| Ubiquinol-8 biosynthesis (prokaryotic) | 211 | -0.0015 | 0.0005 | 0.003 | 0.009 |
| N-acetylneuraminate degradation | 888 | -0.0023 | 0.0008 | 0.003 | 0.009 |
| Fatty acid biosynthesis Initiation (e. coli) | 716 | -0.0031 | 0.0010 | 0.003 | 0.009 |
| L-alanine biosynthesis | 686 | -0.0033 | 0.0011 | 0.002 | 0.009 |
| Menaquinol-11 biosynthesis | 509 | -0.0039 | 0.0013 | 0.002 | 0.009 |
| Menaquinol-12 biosynthesis | 509 | -0.0039 | 0.0013 | 0.002 | 0.009 |
| Menaquinol-13 biosynthesis | 509 | -0.0039 | 0.0013 | 0.002 | 0.009 |
| Phylloquinol biosynthesis | 502 | -0.0023 | 0.0008 | 0.004 | 0.01 |
| Glycolysis, pyruvate dehydrogenase, TCA, and glyoxylate bypass | 343 | -0.0027 | 0.0009 | 0.003 | 0.01 |
| Unstratified superpathway of heme biosynthesis from glutamate | 575 | -0.0023 | 0.0008 | 0.004 | 0.01 |
| Glyoxylate bypass and TCA | 346 | -0.0022 | 0.0008 | 0.005 | 0.01 |
| Heme biosynthesis from glycine | 201 | -0.0012 | 0.0005 | 0.007 | 0.02 |
| Glycol metabolism and degradation | 245 | -0.0014 | 0.0005 | 0.007 | 0.02 |
| Glucose and xylose degradation | 859 | -0.0030 | 0.0011 | 0.008 | 0.02 |
| L-tryptophan biosynthesis | 181 | -0.0027 | 0.0010 | 0.008 | 0.02 |
| Phospholipid biosynthesis I (bacteria) | 886 | -0.0038 | 0.0014 | 0.009 | 0.02 |
| Acetyl-COA biosynthesis | 409 | -0.0015 | 0.0006 | 0.01 | 0.02 |
| Hexitol degradation (bacteria) | 885 | -0.0025 | 0.0010 | 0.01 | 0.03 |
| Guanosine nucleotides de novo biosynthesis I | 906 | -0.0034 | 0.0013 | 0.01 | 0.03 |
| Menaquinol-8 biosynthesis I | 267 | -0.0024 | 0.0010 | 0.01 | 0.03 |
| Demethylmenaquinol-8 biosynthesis | 267 | -0.0021 | 0.0008 | 0.01 | 0.03 |
| L-tyrosine biosynthesis | 337 | -0.0029 | 0.0012 | 0.02 | 0.03 |
| Fatty acids biosynthesis (e. coli) | 270 | -0.0026 | 0.0011 | 0.02 | 0.03 |
| D-glucarate and d-galactarate degradation | 224 | -0.0010 | 0.0005 | 0.02 | 0.04 |
| Fucose and rhamnose degradation | 450 | -0.0024 | 0.0010 | 0.02 | 0.04 |
| Guanosine nucleotides de novo biosynthesis II | 906 | -0.0026 | 0.0012 | 0.03 | 0.049 |

Abbreviation: N means the number of samples (out of the 911 samples) with the species detected.

^1^Analyzed using MaAsLin 2 adjusted for repeated measurements (participant ID as random intercept), age, energy intake, alcohol, smoking, physical activity, using of antibiotics, consumed any probiotics, body mass index at age 21 and fecal sample characteristics.

^2^beta coefficients: relative abundance of super pathways associated with per standard deviation of enterolactone levels, where enterolactone is batch-corrected log-transformed and in unit of per standard deviation; relative abundance of super pathways were standardized and normalized via arc-sin square root transformation. *P*_FDR_: P values after false discovery rate (FDR) correction following the Benjamini–Hochberg method; Table S1 listed all species with a *P*_FDR_ below 0.05.

**Supplementary Table S3 Plasma metabolites significantly associated with enterolactone-predicting species.**

| **Species** | **Metabolites**  **(HMDBID#)** | **Correlation Coefficient** | **Raw**  ***P* value** | ***P*_FDR_** |
| --- | --- | --- | --- | --- |
| Methanobrevibacter smithii | Cinnamoylglycine (HMDB0011621) | 0.35 | <0.001 | <0.001 |
| Methanobrevibacter smithii | Hippurate (HMDB0000714) | 0.27 | <0.001 | <0.001 |
| Methanobrevibacter smithii | Trigonelline (HMDB0000875) | 0.20 | <0.001 | 0.001 |
| Lachnospiraceae bacterium 1 4 56FAA | Cinnamoylglycine (HMDB0011621) | -0.30 | <0.001 | <0.001 |
| Lachnospiraceae bacterium 1 4 56FAA | Hippurate (HMDB0000714) | -0.23 | <0.001 | <0.001 |
| Lachnospiraceae bacterium 1 4 56FAA | N-acetylleucine (HMDB0011756) | 0.23 | <0.001 | <0.001 |
| Lachnospiraceae bacterium 1 4 56FAA | Glycodeoxycholate/glycoche (HMDB0000631) | 0.22 | <0.001 | <0.001 |
| Flavonifractor plautii | Cinnamoylglycine (HMDB0011621) | -0.26 | <0.001 | <0.001 |
| Flavonifractor plautii | Hippurate (HMDB0000714) | -0.24 | <0.001 | <0.001 |
| Flavonifractor plautii | Hydroxycotinine (HMDB0001390) | -0.20 | <0.001 | 0.001 |
| Coprococcus sp. ART55/1 | Cinnamoylglycine (HMDB0011621) | 0.21 | <0.001 | <0.001 |
| Clostridium symbiosum | Cinnamoylglycine (HMDB0011621) | -0.28 | <0.001 | <0.001 |
| Clostridium symbiosum | Hippurate (HMDB0000714) | -0.26 | <0.001 | <0.001 |
| Clostridium symbiosum | N-acetylleucine (HMDB0011756) | 0.21 | <0.001 | <0.001 |
| Clostridium bolteae | N-acetylleucine (HMDB0011756) | 0.24 | <0.001 | <0.001 |
| Clostridium bolteae | Cinnamoylglycine (HMDB0011621) | -0.24 | <0.001 | <0.001 |
| Clostridium bolteae | Hippurate (HMDB0000714) | -0.24 | <0.001 | <0.001 |
| Butyrivibrio crossotus | C18:2 LPC (HMDB0010386) | 0.24 | <0.001 | <0.001 |
| Butyrivibrio crossotus | Trigonelline (HMDB0000875) | 0.22 | <0.001 | <0.001 |
| Butyrivibrio crossotus | Cinnamoylglycine (HMDB0011621) | 0.22 | <0.001 | <0.001 |
| Butyrivibrio crossotus | C18:1 LPC (HMDB0002815) | 0.20 | <0.001 | 0.001 |
| Butyrivibrio crossotus | C18:2 LPE (HMDB0011507) | 0.20 | <0.001 | 0.001 |
| Blautia hydrogenotrophica | Pipecolic acid (HMDB0000070) | -0.28 | <0.001 | <0.001 |
| Blautia hydrogenotrophica | Hydroxycotinine (HMDB0001390) | -0.21 | <0.001 | <0.001 |
| Bacteroides dorei | 2-aminohippuric acid (HMDB0001867) | 0.22 | <0.001 | <0.001 |
| Blautia hydrogenotrophica | 5-acetylamino-6-amino-3-methyluracil (HMDB0004400) | 0.20 | <0.001 | 0.001 |

R^2^: Spearman Partial Correlation adjusted for age, energy intake, alcohol, smoking, physical activity, using of antibiotics, consumed any probiotics, body mass index at age 21 and fecal sample characteristics.
